# Liquiritigenin exerts the anti-cancer role in oral cancer via inducing autophagy-related apoptosis through PI3K/AKT/mTOR pathway inhibition *in vitro* and *in vivo*

**DOI:** 10.1080/21655979.2021.1971501

**Published:** 2021-09-07

**Authors:** Yingchen Ji, Weiwei Hu, Yan Jin, Huiming Yu, Jin Fang

**Affiliations:** a Department of Stomatology, Jiangsu Province Hospital of Chinese Medicine, Jiangsu, 210029, China; b Department of Stomatology, Huai’an Second People’s Hospital and Affiliated Huai’an Hospital of Xuzhou Medical University, Jiangsu, China; c Department of Medical Oncology, Affiliated Huai’an No.1 People’s Hospital, Nanjing Medical University, Jiangsu, China; d Department of Stomatology, Affiliated Huai’an No.1 People’s Hospital, Nanjing Medical University, Jiangsu, China

**Keywords:** Autophagy, apoptosis, oral cancer, oncology, signal pathway, traditional chinese medicine

## Abstract

Operative treatment on oral cancer greatly damages the chewing and language function of the patient, we aim to find better solution with fewer side effects. The anti-tumor effects of Liquiritigenin (LQ) have been explored in kinds of cancers, but not in oral cancer. In this study, our purpose is to reveal the effects of LQ on oral cancer and the associated mechanism.

Cell proliferation was examined through 3-(4,5-Dimethylthiazol-2-yl)-2,5-diphenyltetrazolium bromide (MTT) assay and 5-Ethynyl-2ʹ- deoxyuridine (EDU) staining. Cell apoptosis in cells and tissues were assessed by flow cytometry and terminal dexynucleotidyl transferase-mediated dUTP nick end labeling (TUNEL) staining, respectively. Expressions of AKT and light chain 3 (LC3) were detected through Immunofluorescence. In addition, xenograft model was established by injecting the CAL-27 cells (2 × 10^6^) subcutaneously into the right flanks of mice. Expression of Ki67 and Beclin1 in tissues was valued by Immunohistochemistry (IHC).

We found that cell viability of CAL-27 and SCC-9 was effectively inhibited by LQ. Besides, obvious cell apoptosis and cell autophagy were induced by LQ. In addition, PI3K/AKT/mTOR pathway was sharply inactivated by LQ in oral cancer cells. Corresponding *in vivo* experiments demonstrated that tumor growth was largely restricted, cell apoptosis was augmented and autophagy was enhanced by LQ. What is more, phosphorylation of AKT in tumor tissues could also be inhibited by LQ. LQ inhibited the progression of oral cancer through inducing autophagy-associated apoptosis via PI3K/AKT/mTOR pathway inhibition, revealing a new possible scheme for the treatment of oral cancer.

## Graphical Abstract

**Figure:**
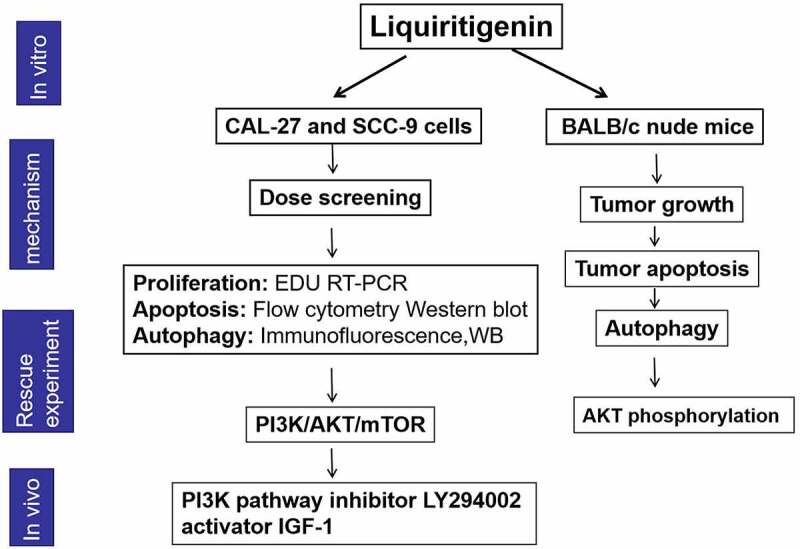

## 1. Introduction

Oral cancer is classified as tongue cancer, pharyngeal cancer, gingival cancer and lip cancer, among which tongue carcinomas accounts for 40% of oral cancers [1,2]. Due to dense distribution of blood vessels and lymph nodes in maxillofacial region and frequent and inevitable local movement, Oral cancer is accompanied by the characteristics of rapid growth and strong invasion [3]. As the main curative treatment for oral cancer now, surgery always brings great damages to the chewing and language function, largely decreasing the quality of life of patients [4]. Therefore, finding better curative methods with fewer side effects is a significant research topic for the basic and clinical research about oral cancer.

Licorice has a long medicinal history for anti-inflammation, anti-vascular diseases, anti-aging, anti-tumor and so on [5,6]. Previous study pointed out that the anti-tumor effects of flavonoids were associated with apoptosis-induction [7,8]. The anti-tumor effects of Liquiritigenin (LQ), a dihydroflavone monomer compound extracted from licorice, have also been studied in kinds of cancers. For example, Di Wang et al. reported that LQ attenuated cytoactive and initiated apoptosis in pituitary adenoma cells [9]. Besides, LQ inhibited cell proliferation, invasion, and epithelial-to-mesenchymal transition in colorectal cancer [10]. However, effects of LQ in oral cancer have never been explored before. Thus, our purpose is to explore the effects of LQ on oral cancer and possible mechanisms.

Autophagy helps the cells adapt to environmental stress by removing damaged or redundant organelles [11]. Through regulating cell metabolism, differentiation, aging and the death, autophagy manages cell remodeling and maintains dynamic balance of cell survival [12]. At the same time, autophagy may act as different roles during tumor development. For example, previous research showed that the defection of autophagy gene beclin1 promoted tumor growth in mice [13]. However, autophagy may also promote tumor growth by keeping mitochondrial function and preserving metabolic homeostasis [14]. At present, research about the Chinese medicine on autophagy in tumor is increasing at home and abroad [15]. For example, quercetin restrained the movement of breast cancer cells by activating AKT-mTOR pathway mediated autophagy [16]. In addition, licochalcone A held-up the progression of osteosarcoma cells through the induction of autophagy and ATM-Chk2 pathway [17]. In addition, autophagy induced by Licorice exerted hepato-protective effects in acute liver injury [18]. However, the association between LQ and autophagy has not been announced before.

In this study, we aimed to explore the effects and possible signal pathway of LQ in oral cancer, aiming to find better treatment solution with fewer side effects. We speculated that LQ may also exert anti-tumor effects in oral cancer like several other proven cancers. We explored the association between LQ and cell proliferation, cell apoptosis and autophagy *in vivo* and *in vitro*, aiming to provide an effective therapeutic strategy for oral cancer treatment.

## 2. Materials and methods

### 2.1 Cell culture and treatment

CAL-27 and SCC-9 cells received from the Chinese Academy of Sciences (Shanghai, China) were cultured in Dulbecco’s modified Eagle’s medium (DMEM) (Gibco, Rockville, MD). The DMEM medium contains 10% fetal bovine serum and is kept in a humidified incubator with 5% CO2 at 37°C.

Liquiritigenin (LQ, Source Leaf Biological Technology Co, LTD, Shanghai, China; Purity > 98.0%) was dissolved in dimethyl sulfoxide (DMSO). The 0.5 mol/L stock solution was sub-packaged at −20°C and was diluted to stated concentration with DMEM before use. The concentration of LQ used in the present study was selected according to previous research [19].

PI3K pathway inhibitor LY294002 (Abcam, Cambridge, UK. 4 µM) and pathway activator IGF-1(Beyotime Biotechnology, Nanjing, China. 10 nM) were used to block or activate the PI3K/AKT/mTOR pathway, respectively. The concentration of LY294002 and IGF-1 was selected according to related reference [20].

## 2.2 3 3-(4,5-Dimethylthiazol-2-yl)-2,5-diphenyltetrazolium bromide (MTT) assay

Cell suspension made from logarithmic cells was inoculated into the 96 well culture plate (3000 cells/well). 12 h later, dissolved LQ solution was added (the final drug concentration was 0, 5, 10, 25, 50, 100, 200, 400, 500, 600 μmol/L, respectively). 24 h later, 20 μL MTT working solution (5 g/L) was incubated with cells in each well for 4 h. Then, 150 μL DMSO was used to dissolve the purple formazan crystals after the supernatant was discarded. At last, the absorbance was measured by a micro-plate reader (Bio-Rad, Hercules, CA, USA) at 490 nm [21].

## 2.3 5-Ethynyl-2ʹ- deoxyuridine (EDU) staining assay

EdU proliferation assay was carried out using the EdU staining proliferation kit (ab222421, Abcam, Cambridge, UK) following the manufacturer’s instructions. Cells were incubated with 50 μM EdU solution for 3 h under optimal growth conditions. Then, fixative solution and permeabilization buffer were added in turn, and each was incubated for 15 min. Soon afterward, the reaction mixture was added to fluorescently label EdU for another 30 min. At last, the fluorescence was observed under fluorescence microscope (Image Systems, Columbia, MD).

### 2.4 Quantitative real-time reverse transcription-polymerase chain reaction (qRT-PCR)

Total RNA in tissues and cells was extracted using TRIzol (Invitrogen, Carlsbad, CA, USA). The concentration and the purity of the RNA were valued on the Nanodrop 2000 c ultra micro spectrophotometer (Thermo Fisher Scientific, Massachusetts, USA). The cDNA was obtained from reverse transcription using the PrimeScript RT reagent Kit (Takara Biotechnology, Dalian, China). The qRT-PCR was performed using SYBR Green dye (Invitrogen). The primers for Ki67 and proliferating cell nuclear antigen (PCNA) were obtained from Vazyme Biotech (Jiangsu, China). The primers were listed as follows: Ki67, (forward: 5'-CTCCATCCTGGCCTCGCTGT-3') and (reverse: 5'- GCTGTCACCTTCACCGTTCC −3'); PCNA, (forward: 5'-GTAATGACTCTATGTGATGCC-3') and (reverse: 5'-GATAAAAGGTTACAAACGATG-3'). All reactions were performed at least for three times and the data were quantified using the 2^−ΔΔCt^ method [22].

### 2.5 Flow cytometry

CAL-27 and SCC-9 cells pretreated with different concentration of LQ were harvested and prepared for cell apoptosis detection. The cells were blended with Annexin V-FITC and PI (Sigma, St. Louis, MO, USA) for 15 min at room temperature in dark. Then, the stained cells were analyzed through flow cytometry [23].

### 2.6 Western blot

The western blot was conducted following the standard procedure [24]. Total proteins in collected cells were extracted and the concentration of protein was checked using the BCA™ Protein Assay Kit (Thermo Fisher Scientific, Massachusetts, USA). Equivalent protein samples were separated by polyacrylamide gel electrophoresis and the protein was then transferred onto the polyvinylidene difluoride membranes (Millipore, Bedford, MA, USA). The primary antibodies (Abcam, Cambridge, UK) against the following proteins were listed: caspase-3 (1:1,000, ab52293), cleaved-caspase-3 (1:1,000, ab13585), caspase-9 (1:1,000, ab25758), cleaved-caspase-9 (1:1,000, ab32068), light chain 3 (LC3)I (1:1,000, ab12273), LC3II (1:1,000, ab48394), ATG7 (1:1,000, ab42373), Beclin1 (1:1,000, ab62557), PI3K (1:1,000, ab189403), p-PI3K (1:1,000, ab182651), AKT (1:1,000, ab18785), p-AKT (1:1,000, ab8933), mTOR(1:1,000, ab2573), p-mTOR(1:1,000, ab7683) and actin (1:1,000, ab62346). After washed for at least three times using phosphate buffer solution containing 0.05% Tween-20, the membranes were incubated with corresponding secondary antibody (1:10,000, Santa Cruz, CA, USA) at room temperature for 2 h. After washed, the membranes were developed using ECL. The protein intensity was quantified on the Bio-Rad Image Lab™ 3.0 version software (Bio-Rad Laboratories).

### 2.7 Immunofluorescence

After fixed and permeabilized, the cells were incubated with specific antibody against LC3 and AKT (Abcam, Cambridge, UK) at 4°C overnight. Then, FITC-conjugated secondary antibody (1:1000, sc-69,872, Santa Cruz Biotechnology, CA, USA) was incubated with the cells at 37°C for 1 h in dark. Fluorescence was measured on confocal laser scanning microscopy (Carl Zeiss LSM 880, Germany) [25].

### 2.8 Establishment of oral cancer xenograft model

Animal experiments here were ratified by the Animal Care and Use Committees of Nanjing Medical University affiliated Huai’an No.1 People’s Hospital. The study was conducted following the principles of the Declaration of Helsinki. CAL-27 cells (2 × 10^6^) were injected into the right flanks of 5-week-old female BALB/c nude mice subcutaneously to establish the xenograft model. Then the mice were divided into the control group and the observation group. The mice in the observation group received 20 mg/kg/day Liquiritigenin through oral gavage and mice in the control group adopted equal amount of DMSO. The mice were fed in standard laboratory with free access to food and water. The tumor volumes were calculated every 5 days post injection. Tumor volumes (mm^3^) = length × width^2^/2. 30 days after treatment, the mice were sacrificed through rapid cervical dislocation. Tumor was excised, tumor weight was valued and tumor tissues were prepared for further detection.

### 2.9 Immunohistochemistry (IHC)

The tumor tissues were fixed, embedded and made into 4-μm sections. Then the sections were incubated with primary antibodies for the proliferation marker protein Ki67 (anti-Ki67) (Cell Signaling Technology, Boston, USA) and Beclin1 (anti-Beclin1) (Cell Signaling Technology, Boston, USA). After incubated with corresponding secondary antibody (BOSTER Biological Technology, Wuhan, China), tissue sections were stained with 3, 3′-diaminobenzidine and hematoxylin in turn. After dehydrated and mounted, the tissues were observed under the fluorescence inverted microscope [26].

### 2.10 Terminal dexynucleotidyl transferase-mediated dUTP nick end labeling (TUNEL) staining

After fixed with 4% paraformaldehyde for 30 min, the tissue sections were washed. Afterward, the tissues sections were incubated with 0.3% Triton-X 100 for 5 min and 50 μL TUNEL reaction buffer for 1 h, respectively. The nucleus was stained with hematoxylin for 1 min at room temperature without light. At last, cell apoptosis in the tissues was visualized using Laser scanning confocal microscope (LSM 710, Carl Zeiss, Germany) [24].

### 2.11 Statistical analysis

All experiments were conducted at least for 3 times. All the data were shown with the form of mean ± standard deviation. GraphPad 6.0 software (GraphPad, San Diego, CA, USA) was adopted for statistical analysis. P values were calculated using the one-way analysis of variance followed by Bonferroni post hoc test. P < 0.05 was deemed as statistically significant.

## Results

In this study, we explored the effects of LQ on cell proliferation, cell apoptosis and cell autophagy, aiming to learn about the anti-tumor effects of LQ in oral cancer. Besides, we probed into the associated signalpathway and conducted the demonstration test *in vivo*.

## Liquiritigenin (LQ) suppresses cell proliferation in oral cancer cells

**Figure 1.**
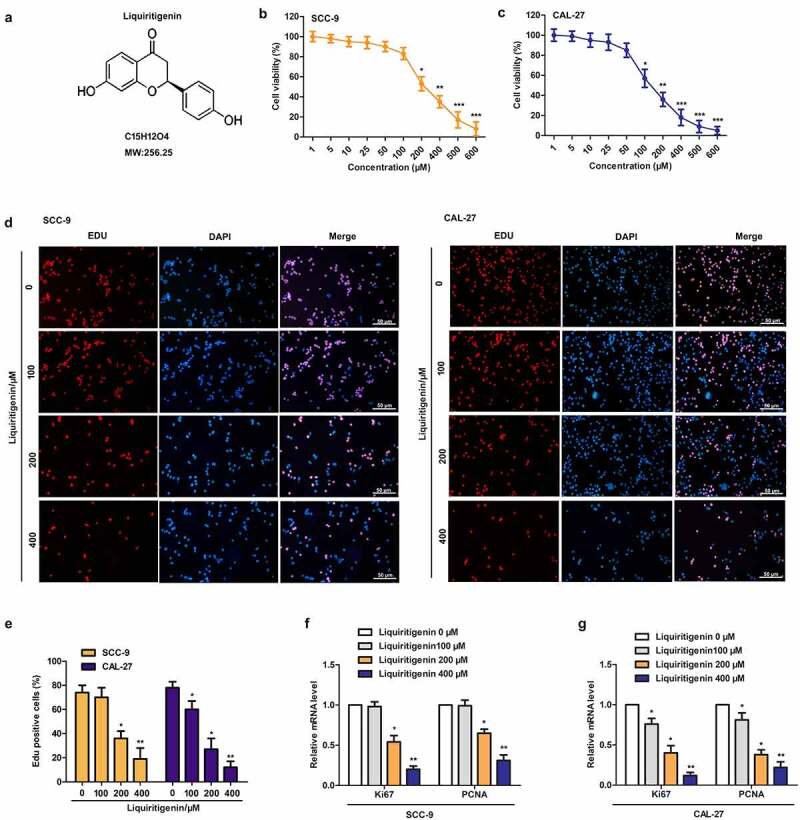
**Liquiritigenin (LQ) suppresses cell proliferation in oral cancer cells**. A. Chemical structure of Liquiritigenin (LQ). B-C. CAL-27 and SCC-9 cells were treated with a series of LQ ranging from 0–600 µM for cell viability detection through MTT assay. D. EDU staining was conducted to detect the proliferation of CAL-27 and SCC-9 cells. The nuclei of in proliferative and DNA replication state were red fluorescence. DAPI stained nuclei were blue fluorescence (scale: 50 μm). E. The statistical results of EDU positive cells (10 random fields were selected). F-G. Relative expression of Ki67 and PCNA was detected through qRT-PCR. *P < 0.05, **P < 0.01 VS LQ 0 µM group

Chemical structure of Liquiritigenin was exhibited as shown in Figure1A. CAL-27 and SCC-9 cells were treated with LQ ranging from 0–600 µM. Results of MTT assay in Fig.1B-1 C showed that cell viability was effectively inhibited by LQ in dose dependent manner. In order to better screen effective dose of LQ, four doses (0, 100, 200, 400 μm) of LQ were selected for the follow-up experiments. The results of EDU staining showed that the nuclei of CAL-27 and SCC-9 cells showed red fluorescence in proliferative and DNA replication state, and those of DAPI stained nuclei were blue fluorescence. We could clearly found that the red fluorescence was gradually weakened with increasing dose of LQ (Fig.1D) in CAL-27 and SCC-9 cells, exactly as the statistical results of EDU positive cells in Fig.1E (10 random fields were selected). At the same time, relative expression of cell proliferation related Ki67 and PCNA in SCC-9 and CAL-27 cells was also largely suppressed by LQ, and significant difference was appeared in 200 μm and 400 μm groups (Fig.1F-1 G). Thus, proliferation of oral cancer cells is e effectively suppressed by LQ when the concentration of LQ was larger than 200 μm.

## Liquiritigenin induces cell apoptosis in oral cancer cells

**Figure 2.**
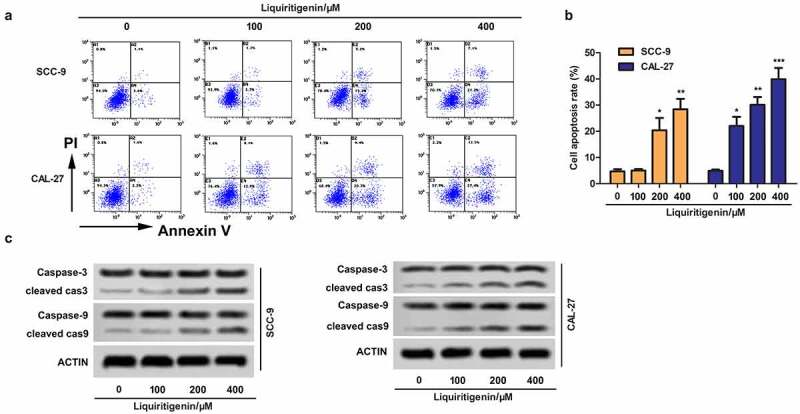
**Liquiritigenin induces cell apoptosis in oral cancer cells**. A. Effects of LQ on cell apoptosis were also valued through flow cytometry. B. Specific apoptosis rate comparison was also showed. C. Expression of cell apoptosis related proteins cleaved-caspase-3/caspase-3 and cleaved-caspase-9/caspase-9 was examined through western blot. *P < 0.05, **P < 0.01, ***P < 0.001 VS LQ 0 µM group

Effects of LQ on cell apoptosis were also valued. The results showed that cell apoptosis was enhanced with increasing concentration of LQ (Fig.2A–2B). The expression of cleaved-caspase-3/caspase-3 and cleaved-caspase-9/caspase-9 was examined through western blot (Fig.2C). The results showed that the expression of cleaved-caspase-3 and cleaved-caspase-9 was strongly up-regulated by LQ, especially in the 200 μm and 400 μm group (the Table 1). Thus, cell apoptosis of oral cancer cells is induced by LQ with a concentration larger than 200 μm.

**Table 1.** Quantitative analysis about the expression of cleaved cas3/cas3 and cleaved cas9/cas9 detected by western blot in Figure2C

Group	SCC-9		CAL-27	
	cleaved cas3/cas3	cleaved cas9/cas9	cleaved cas3/cas3	cleaved cas9/cas9
LQ 0 µM	0.02 ± 0.01	0.01 ± 0.01	0.02 ± 0.01	0.01 ± 0.01
LQ 100 µM	0.02 ± 0.01	0.01 ± 0.01	0.07 ± 0.03*	0.06 ± 0.02*
LQ 200 µM	0.26 ± 0.05**	0.18 ± 0.04**	0.26 ± 0.05**	0.29 ± 0.04**
LQ 400 µM	0.44 ± 0.06**	0.46 ± 0.07**	0.65 ± 0.07**	0.42 ± 0.04**

*P < 0.05, **P < 0.01 VS LQ 0 µM group.

## Liquiritigenin induces obvious cell autophagy in oral cancer cells

**Figure 3.**
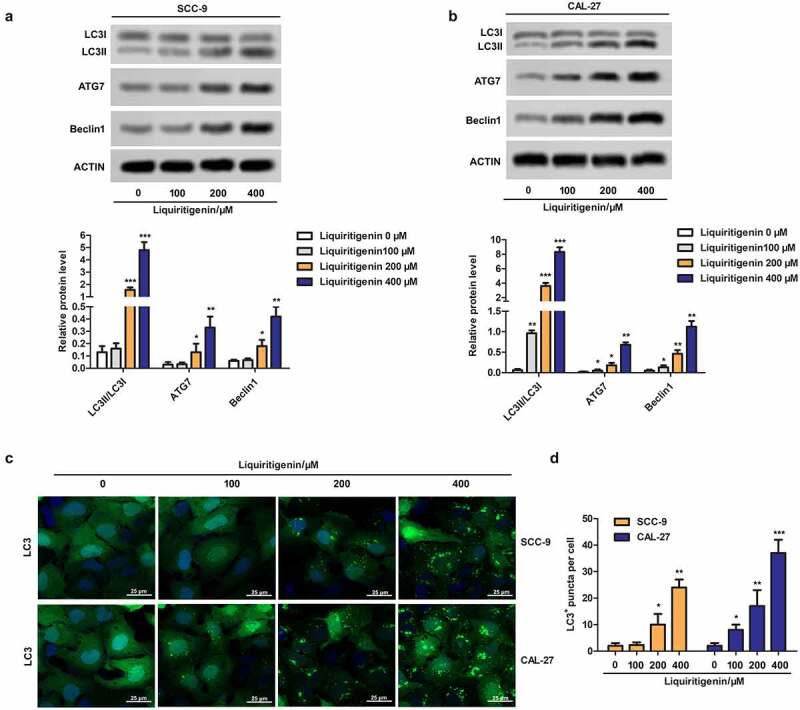
**Liquiritigenin induces obvious cell autophagy in oral cancer cells. A-B**. Expressions of autophagy related LC3II/LC3I, ATG7 and Beclin1 in CAL-27 and SCC-9 cells were detected through western blot. C. LC3^+^ puncta were observed through immunofluorescence. Magnification: 200 × . D. Number of LC3^+^ puncta in cells in different groups was counted and showed. *P < 0.05, **P < 0.01, ***P < 0.001 VS LQ 0 µM group

To further investigate the possible mechanism related to LQ induced apoptosis, cell autophagy was checked here. As shown in Fig.3A-3B, the expressions of LC3II/LC3I, ATG7 and Beclin1 were detected. The results showed that LQ could markedly promote the transformation from LC3I to LC3II and up-regulate the expression of ATG7 and Beclin1when the concentration of LQ was larger than 200 μm. In addition, LC3^+^ puncta were observed through immunofluorescence and the results in Fig.3C clearly showed that number of LC3^+^ puncta was gradually increased with increasing concentration of LQ. The number of LC3^+^ puncta in different groups was counted and was showed in the form of a bar chart (Fig.3D). Thus, obvious cell autophagy is induced by LQ and we speculate that LQ induces obvious cell apoptosis through enhancing autophagy.

## Liquiritigenin inactivates the PI3K/AKT/mTOR pathway in oral cancer cells

**Figure 4.**
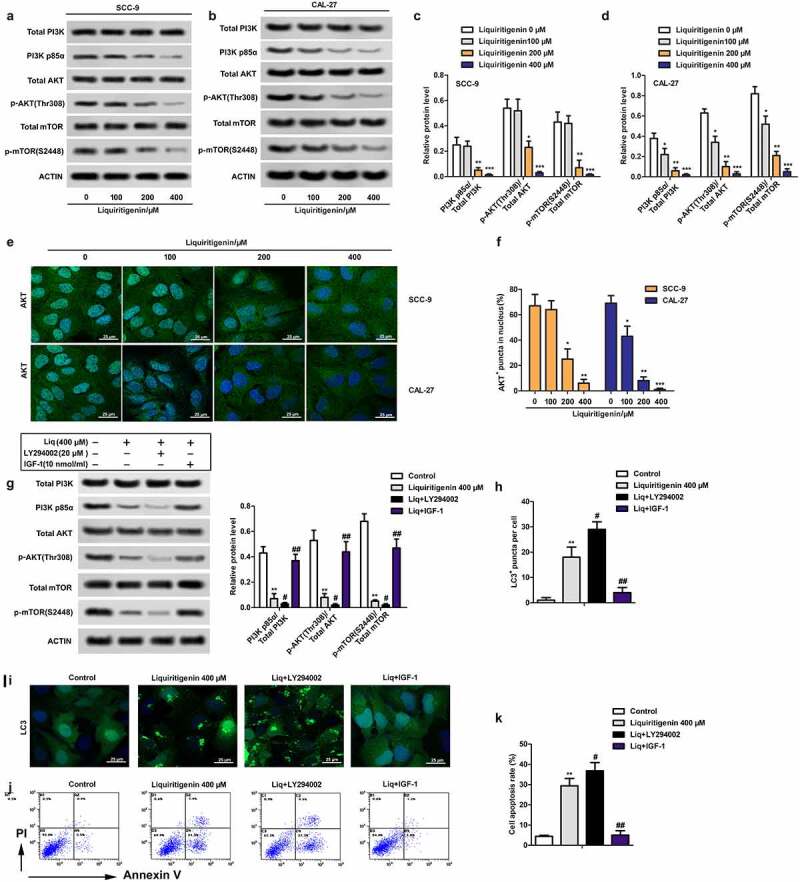
**Liquiritigenin inactivates the PI3K/AKT/mTOR pathway in oral cancer cells**. A-D. Expressions of PI3K/AKT/mTOR pathway related PI3K p85α/ total PI3K, p-AKT (Thr308)/total AKT and p-mTOR (S2448)/ total mTOR were detected through western blot in CAL-27 and SCC-9 cells. E. Immunofluorescence was conducted to observe the transport of AKT. Magnification: 200 × F. Statistical results showed the number of cells with AKT fluorescence in nucleus. G. PI3K pathway inhibitor LY294002 (4 μM) and pathway activator IGF-1(10 nM) were used to block or activate the PI3K/AKT/mTOR pathway. Expressions of related proteins were detected through western blot. H. Number of LC3^+^ puncta in cells in different groups was counted and showed. I. LC3 fluorescence in cells was observed through immunofluorescence. Magnification: 200 × J. Cell apoptosis was valued through flow cytometry. K. Specific apoptosis rate comparison was showed. A-F: *P < 0.05, **P < 0.01, ***P < 0.001 VS LQ 0 µM group. G-K: *P < 0.05, **P < 0.01, ***P < 0.001 VS control group

The activation of PI3K/AKT/mTOR pathway has been commonly considered as a sign of tumor development and deterioration. In our study, we found that the rate of PI3K p85α/ total PI3K, p-AKT (Thr308)/total AKT and p-mTOR (S2448)/ total mTOR was all largely suppressed by LQ in dose dependent manner (Fig.4A-4D). Besides, immunofluorescence showed that LQ promoted the transfer of AKT from nucleus to cytoplasm (Fig.4E). Statistical result in Fig.4F made it more visually apparent that the number of cells with AKT fluorescence in nucleus decreased with increasing concentration of LQ. In order to further verify the role of PI3K/AKT/mTOR pathway in the process, PI3K pathway inhibitor LY294002 (20 μM) and pathway activator IGF-1(10 nM/L) were used to block or activate the PI3K/AKT/mTOR pathway (Fig.4G). Accordingly, we found that the inhibition effects of LQ on the PI3K/AKT/mTOR pathway, autophagy and apoptosis were enhanced by LY294002. Of course, the effects of LQ on the signal pathway, autophagy and apoptosis were counteracted by the addition of IGF-1 (Fig.4H-K). Thus, the PI3K/AKT/mTOR pathway in oral cancer cells is inactivated by high-dose LQ.

## Liquiritigenin inhibits tumor growth, induces cell apoptosis and autophagy and inactivates the PI3K/AKT/mTOR pathway *in vivo*

**Figure 5.**
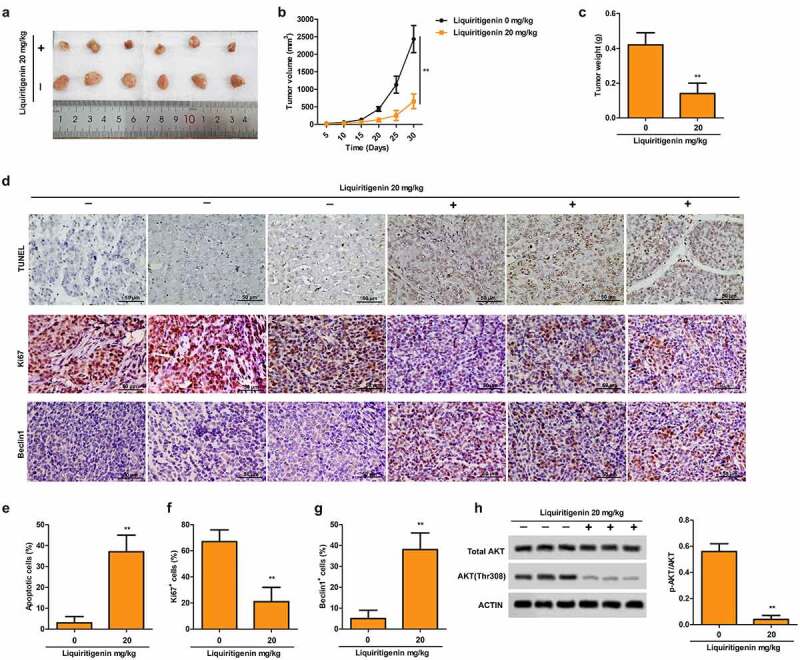
**Liquiritigenin inhibits tumor growth, induces cell apoptosis and autophagy and inactivates the PI3K/AKT/mTOR pathway *in vivo***. Oral cancer xenograft model was established by injecting with CAL-27 cells. The mice in the experimental group received liquiritigenin 20 mg/kg/day through oral administration. A-C. Representative pictures (a) of tumor, tumor growth curve (b) and tumor weight (c) in each group were shown. D. TUNEL staining (upper) showed the proportion of apoptotic cells (scale: 50 μm). Expression of Ki67 (middle) and Beclin1 (lower) in tissues was valued through immunohistochemistry (scale: 50 μm). E-G. Statistical results about proportion of apoptotic cells, Ki67^+^ cells and Beclin1^+^ cells. H. Phosphorylation of AKT in tissues was also detected through western blot. **P < 0.01VS LQ 0 µM group

Representative pictures in Fig.5A and tumor growth curve in Fig.5B showed that tumor growth was largely suppressed by LQ with the dose of 20 mg/Kg. At the same time, the LQ group had significantly lighter tumor weight than the control group (Fig.5C). In addition, pictures of TUNEL staining (Fig.5D, upper) and the statistical results in Fig.5E showed that proportion of apoptotic cells was obviously elevated by LQ. Results of immunohistochemistry for Ki67 (Fig.5D, middle) and Beclin1 (Fig.5D, lower) and corresponding statistical results (Fig.5F-Fig.5G) showed suppressed expression of Ki67 and elevated expression of Beclin1 induced by LQ, indicating that cell proliferation ability was suppressed and cell autophagy was enhanced in vivo. What is more, phosphorylation of AKT was also inhibited by LQ as shown in Fig.5H. Thus, LQ inhibits tumor growth, induces cell apoptosis and autophagy and inactivates the PI3K/AKT/mTOR pathway *in vivo*.

These above results demonstrated that LQ exerted anti-tumor effects in oral cancer through inhibiting cell proliferation and promoting cell apoptosis and autophagy at the same time. Besides, we speculated that the anti-tumor effects of LQ were associated with the inactivation of PI3K/AKT/mTOR pathway.

## Discussion

Tumor is commonly accompanied by unbalanced cell proliferation and cell apoptosis. Apoptosis, also called as programmed cell death, is the process during which cells voluntarily end their lives following gene regulation [27]. Thus, apoptosis induction has become a new anti-tumor research direction, which is of great significance in clinical treatment [28]. Herein, the apoptotic effects of liquiritigenin (LQ) in oral cancer and the related mechanism were investigated.

Traditional Chinese Medicine has shown certain curative effects in anti-tumor research, with the advantages of lower toxicity and fewer side effects compared with chemical drugs [29]. LQ is a kind of dihydroflavone monomer extracted from licorice. According to previous studies, LQ induced apoptosis through promoting the production of reactive oxygen species, which is accompanied by the change of mitochondrial membrane potential and antioxidant enzyme activity [9]. Besides, LQ may induce apoptosis by elevating Bax and suppressing Bcl-2 to sensitize caspase-9 and caspase-3 [30]. High recurrence rate and high mortality rate of malignant tumors are commonly induced by rapid proliferation of tumor cells. In our study, viability of CAL-27 and SCC-9 cells was actively suppressed by LQ when the concentration of LQ was larger than 200 μm, which is conform to the research of Shi-ping Zhang et al. in human hepatoma SMMC-7721 cells [31]. We clearly found that red fluorescence which indicated proliferation and DNA replication was gradually weakened with increasing dose of LQ. At the same time, the expression of Ki67 and PCNA was both largely decreased by LQ. Besides, obvious cell apoptosis was induced by LQ, especially when the dose was greater than 200 μm. The dosage of the drug and the concentration of its active ingredients in the blood have direct relationship. Thus, more repeated clinical experiments should be conducted to determine the most appropriate dosage of traditional Chinese medicine.

Damaged organelles and macromolecules in cells were degraded in the process of autophagy [32]. At first, autophagy was considered as a self-protection mechanism for cell survival through clearing damaged organelles and resisting pathogen infection [33]. However, more and more evidence shows that autophagy also regulates the occurrence and development of tumors [34]. At the same time, related research revealed that autophagy might play completely opposite roles during the progression of tumor. For example, enhanced autophagy helps the cells to survive the micro-environmental stress and increase the growth and aggressiveness of cancer cells in the early stage [35]. However, autophagy may play an anti-tumor role through inducing cell apoptosis in the late stage [36]. At present, autophagy has become a promising target of tumor research and treatment [37]. Besides, Licorice was reported to induce apoptosis and autophagy in gastric cancer cells both *in vitro* and *in vivo* [38]. However, direct association between LQ and autophagy has never been explored before. In our study, LQ induced obvious cell autophagy in CAL-27 and SCC-9 cells, presenting as enhanced LC3-II accumulation and elevated expression of ATG7 and beclin1. Thus, we speculated that LQ exerted anti-tumor effects through inducing autophagy. As an important component during autophagy, ATG7 involves the Atg12 and the Atg8 conjugation system [39]. Previous study has also shown that Danggui Buxue Tang enhanced autophagy-related death of colorectal cancer cells through stimulating the production of Atg7 [40].Similarly, ka-sai-ping, a Traditional Chinese Medicine, exerted anti-tumor effects by enhancing autophagy with beclin1 induction [41]. The effects of traditional Chinese medicine on tumor have multi-targets and multi-channels. There may also be other mechanism related to LQ in oral cancer, but autophagy is still a focus of research due to its importance in cancer progression.

Just as Weiping Liu et al. reported, betulinic acid acted on hepatocellular carcinoma through enhancing apoptosis via inactivating the PI3K/AKT/mTOR pathway [42]. In our study, we also investigated associated signal pathway through which LQ exerted anti-tumor effects. PI3K/AKT/mTOR pathway has been considered as a prospective target for tumor therapy. Previous study pointed out that LQ inhibited cell proliferation, invasion, and epithelial-to-mesenchymal transition in HCT116 cells via inactivating the PI3K/AKT pathway [10]. In our present study, LQ obviously suppressed the phosphorylation of PI3K, AKT and mTOR. Besides, immunofluorescence assay also showed that LQ promoted the transfer of AKT from nucleus to cytoplasm, thus inactivating the PI3K/AKT/mTOR pathway. Activation of AKT is an important feature of malignant tumors [43] and it is positively correlated with the poor prognosis and drug resistance [44]. At the same time, the induction of p-mTOR is closely related to tumor invasion, tumor stage and low survival [45]. To further verify our conclusion, PI3K pathway inhibitor LY294002 and PI3K pathway activator IGF-1 were used in the study to further block or strengthen the PI3K pathway as previously described [46,47].The effects of high-dose LQ on the signal pathway, autophagy and apoptosis were all enhanced by LY294002 and were counteracted by the addition of IGF-1. Thus, we conjecture that LQ exerted anti-tumor effects through inactivating the PI3K/AKT/mTOR pathway.

At last, we also conducted corresponding *in vivo* experiments. The *in vivo* experiments also showed that LQ effectively suppressed tumor growth, induced cell apoptosis and enhanced autophagy. Just as Yuxin Liu et al. reported, LQ effectively restrained the growth of tumor in a mouse model established by injecting with HeLa cells [48]. Besides, LQ also suppressed the phosphorylation of AKT in tumor progression. Thus, anti-tumor effects of LQ were also associated with the inactivation of PI3K/AKT/mTOR pathway and autophagy-related apoptosis *in vivo*.

## Limitation

At present, this experiment only explored the anti-cancer of LQ at the cellular and animal levels. Besides, we only valued the effects of LQ on cell proliferation, apoptosis and autophagy, the effects of LQ on cell mobility will be further explored in our following study. In addition, lots of experiments about the effective dose and drug toxicity are still needed for further clinical application.

## Conclusion

Taken together, our findings demonstrated that LQ inhibited the progression of oral cancer cells by inducing autophagy-associated cell apoptosis via the inactivation of PI3K/AKT/mTOR pathway, revealing a novel mechanism through which LQ acts as a natural drug in oral cancer treatment. In our following study, we will explore the effects of LQ on cell mobility. Besides, more associated signal pathways will also be explored.

## Supplementary Material

Supplemental Material

## Highlights

1. LQ suppresses cell proliferation in oral cancer cells.
2. LQ induces cell apoptosis and autophagy in oral cancer cells.
3. LQ inactivates the PI3K/AKT/mTOR pathway in oral cancer cells.
4. LQ exerts anti-tumor effects *in vivo*.

## Abbreviations

LQ, Liquiritigenin; ROS, reactive oxygen species; DMEM, Dulbecco’s modified Eagle’s medium; DMSO, dimethyl sulfoxide; LC3, light chain 3; PCNA, proliferating cell nuclear antigen; IHC, Immunohistochemistry.
