# Supplementary figures and images for: Liquiritigenin exerts the anti-cancer role in oral cancer via inducing autophagy-related apoptosis through PI3K/AKT/mTOR pathway inhibition in vitro and in vivo
Source: Bioengineered. 2021 Sep 7;12(1):6070–82. doi: 10.1080/21655979.2021.1971501 (PMC8806794; doi:10.1080/21655979.2021.1971501)

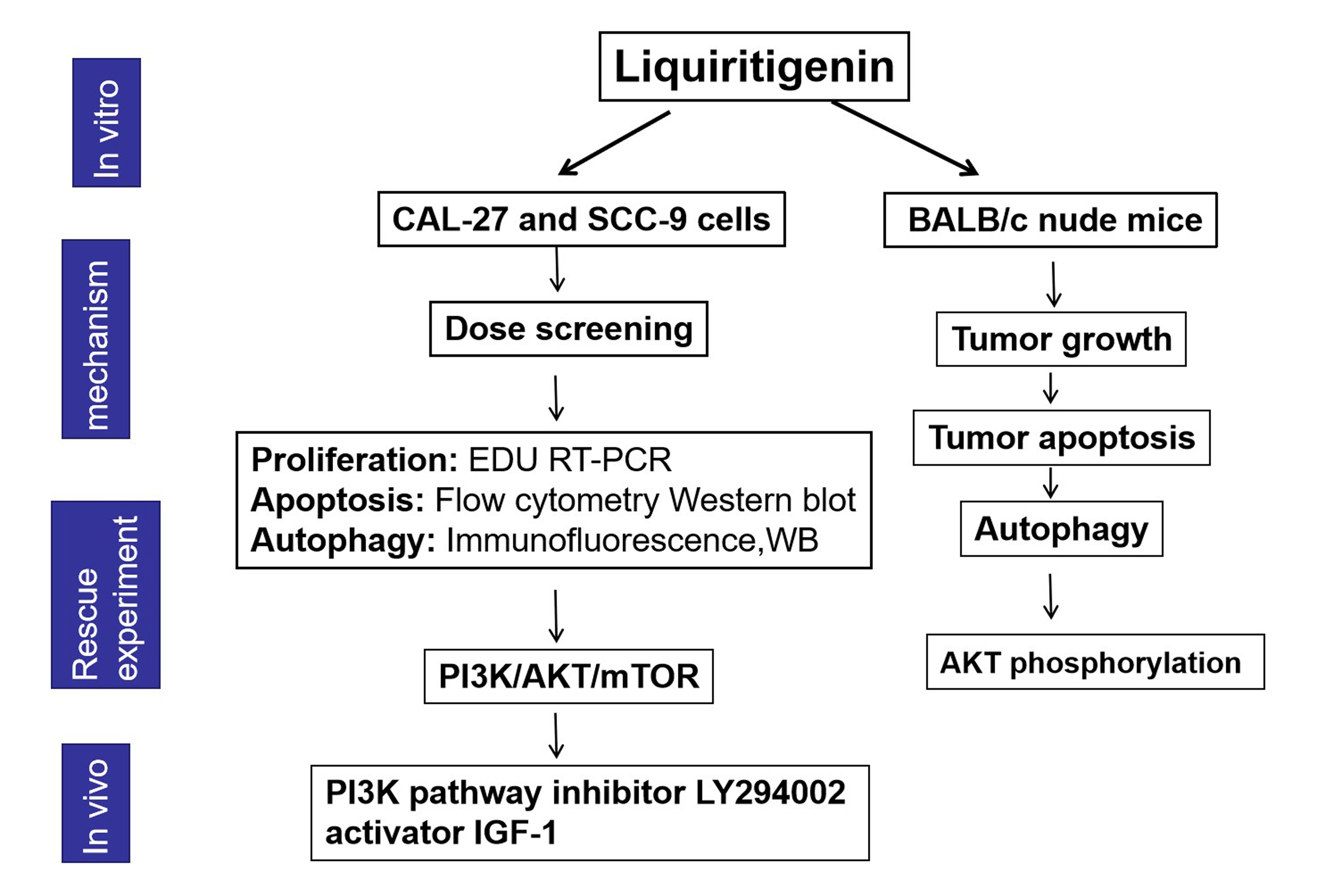

Supplement: Supplemental Material [file KBIE_A_1971501_SM8595.tif]
